# Prospective study using artificial neural networks for identification of high-risk COVID-19 patients

**DOI:** 10.1038/s41598-025-00925-3

**Published:** 2025-05-23

**Authors:** Mateo Frausto-Avila, Roberto de J. León-Montiel, Mario A. Quiroz-Juárez, Alfred B. U’Ren

**Affiliations:** 1 Centro de Física Aplicada y Tecnología Avanzada, Universidad Nacional Autónoma de México, Boulevard Juriquilla 3001, 76230 Querétaro, Mexico; 2 Instituto de Ciencias Nucleares, Universidad Nacional Autónoma de México, Apartado Postal 70-543, 04510 Mexico, CDMX Mexico

**Keywords:** Machine learning, neural networks, COVID-19, Computational models, Data processing, Machine learning, Public health, Computational science

## Abstract

The COVID-19 pandemic caused a major public health crisis, with severe impacts on global health and the economy. Machine learning (ML) has been crucial in developing new technologies to address challenges posed by the pandemic, particularly in identifying high-risk COVID-19 patients. This identification is vital for efficiently allocating hospital resources and controlling the virus’s spread. Comprehensive validation of these intelligent approaches is necessary to confirm their clinical usefulness and help create future strategies for managing viral outbreaks. Here we present a prospective study to evaluate the performance of state-of-the-art ML models designed to identify high-risk COVID-19 patients across four clinical stages. Using artificial neural networks trained with historical patient data from Mexico, we assess the models’ accuracy across six epidemiological waves without retraining them. We then compare their performance against neural networks trained with cumulative historical data up to the end of each wave. The findings reveal that models trained on early data can effectively predict high-risk patients in later waves, despite changes in vaccination rates, viral strains, and treatments. These results suggest that artificial intelligence-based patient classification methods could be robust tools for future pandemics, aiding in predicting clinical outcomes under evolving conditions.

## Introduction

A report of several cases of viral pneumonia by the Wuhan Municipal Health Commission in China on 12 December 2019 evolved into the declaration of the COVID-19 pandemic by the World Health Organization on 11 March 2020^1,2^. The profound impact of COVID-19 on a global scale is attributed to its highly contagious nature and substantial mortality rate. This infectious disease has not only inflicted a severe toll on the global population but has also imposed immense challenges on governments and the world economy^3,4^. The ensuing strain has compelled nations to navigate unprecedented difficulties in their efforts to contain the virus and mitigate its far-reaching consequences. The crisis has underscored the inadequacies of healthcare systems globally, revealing critical deficiencies in hospital equipment, medical personnel, and overall healthcare infrastructure. This has served as a testament to the systemic vulnerabilities and the urgent need for comprehensive emergency preparedness and response strategies on a global scale^5–7^.

Several different strategies have been explored to tackle challenges associated with logistics, supply chain management, and the mathematical modeling of viral spread^8–16^. These endeavors have been aimed at preemptively addressing or alleviating deficiencies in emergency-response systems and healthcare infrastructures. In particular, the identification of high-risk patients is important because hospital resources and capacities must be adequately managed to prevent the collapse of healthcare systems^17^. In this direction, several approaches based on machine-learning algorithms have been proposed to identify, from the earliest stage possible, patients who are likely to become ill or critically ill. These approaches make predictions relying on basic patient information^18–23^, clinical symptoms^24,25^, as well as travel history^26^ and the discharge time of hospitalized patients^27^. Some other efforts focus on identifying patients that require specialized care, namely hospitalization and/or special care units^28,29^, or patients at a higher fatality risk^30^.

**Fig. 1:**
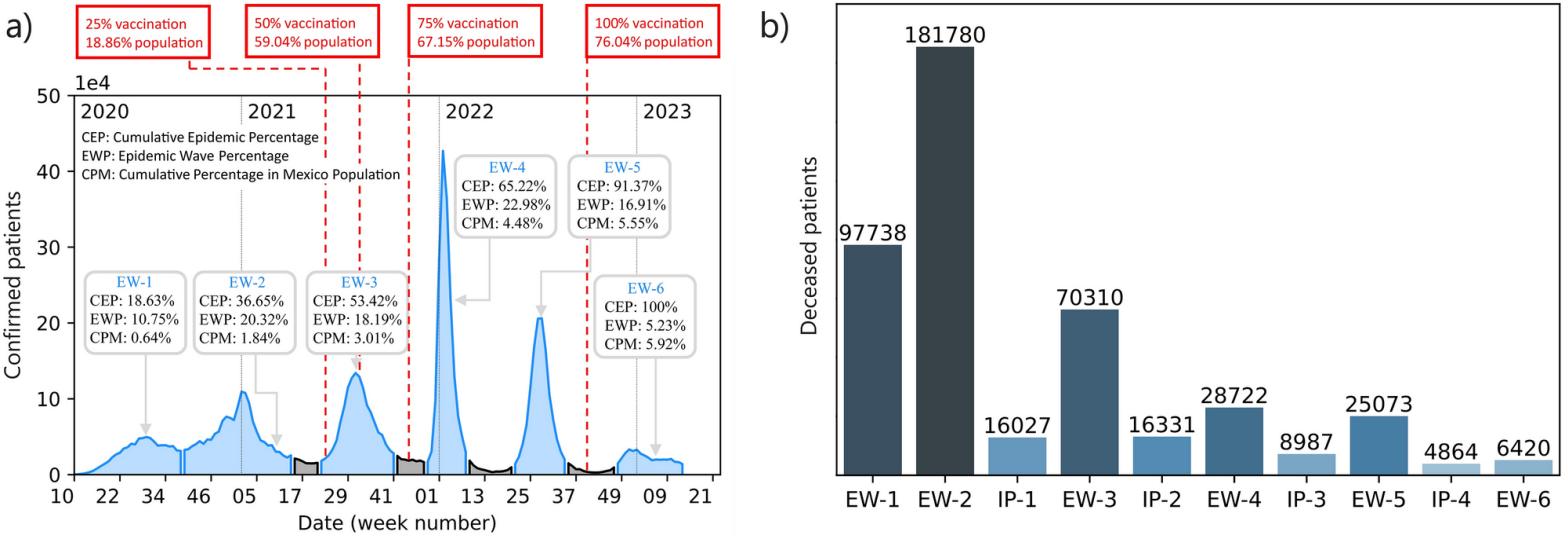
**(a)** Epidemic curve of confirmed cases in Mexico from week 10 of 2020 to week 13 of 2023, spanning a total of 162 weeks. Blue regions correspond to epidemiological waves (EW), while gray regions indicate inter-pandemic periods (IP). Additionally, vaccination records for persons who received at least one dose of complete treatment are indicated in the red rectangles. The initials used in insets correspond to CEP: cumulative epidemic percentage, EWP: epidemic wave percentage, and CPM: cumulative percentage in the Mexican population. **(b)** Recorded deaths in Mexico during the COVID-19 outbreak.

Further studies have been reported that investigate the dynamics of epidemiological waves, thus addressing the complexity of viral spread^31,32^. Despite notable advances in the development of machine learning-based algorithms for the early identification of high-risk patients^33^, it is crucial to highlight the importance of conducting prospective studies that validate the effectiveness and accuracy of these algorithms in real-world situations. The application of prospective approaches will thus significantly contribute to the evaluation of the clinical utility of such tools, thus ensuring their reliability in practical settings for the improvement of medical decision-making during health emergencies. The successful implementation of such tools may improve resource management in hospitals and healthcare units in the event of future health crises.

In this work, we present a prospective validation study using very compact machine-learning models that we proposed in Ref.^17^, designed for the identification of high-risk COVID-19 patients, across four clinical stages, in Mexico. Our study is based on a patient database, publicly made available by the Mexican federal government, covering the period from the 10th week of 2020 to the 13th week of 2023, which includes (i) demographic, (ii) COVID-19 status, and (iii) comorbidity information for patients known or suspected to have been infected with COVID-19, as reported from within the Mexican healthcare system. Importantly, the treatment outcome (i.e. recovery or death) is available for each patient on this database. To evaluate the predictive performance of our models through the six COVID-19 epidemiological waves identified in Mexico, we determine the algorithm accuracy for patients within each wave without any retraining of the neural networks reported in Ref.^17^. Subsequently, we compare their effectiveness against neural networks trained with cumulative historical data, covering the period up to the end of each wave. Our results indicate that models trained with early historical data exhibit strong predictive capabilities throughout all subsequent epidemiological waves. This demonstrates that artificial intelligence algorithms can not only provide accurate identification of high-risk patients based on limited data, but may be robust despite a constantly evolving set of conditions, particularly in terms of the population vaccination status, the dominant viral strains, and the available medical treatments. This paper is structured as follows: section “Materials and methods” describes the database used for this study, section “Methodology” presents a detailed description of our findings, and section “Results” is devoted to our conclusions.

## Materials and methods

### Data

The prospective validation study was conducted using the publicly available database of COVID-19 patients in Mexico. This database, which includes all officially reported confirmed and suspected cases of COVID-19 in Mexico, is available in the Statistical Yearbook of Disease (Anuario Estadístico de Morbilidad) published by the General Epidemiological Council (Dirección General de Epidemiología) that is part of the Ministry of Health (Secretaría de Salud) of the Federal Government of Mexico^34^.

**Table 1:** Epidemiological waves (EW) and inter-pandemic periods (IP) in Mexico from the 10th week of 2020 to the 13th week of 2023.

Event	Period (weeks)	Duration (weeks)
EW-1	20 to 39 of 2020	31
EW-2	39 of 2020 to 15 of 2021	28
IP-1	15 to 22 of 2021	07
EW-3	22 to 42 of 2021	20
IP-2	42 to 50 of 2021	08
EW-4	50 of 2021 to 09 of 2022	11
IP-3	09 to 21 of 2022	12
EW-5	21 to 33 of 2022	12
IP-4	33 to 48 of 2022	15
EW-6	48 of 2022 to 13 of 2023	18

**Fig. 2:**
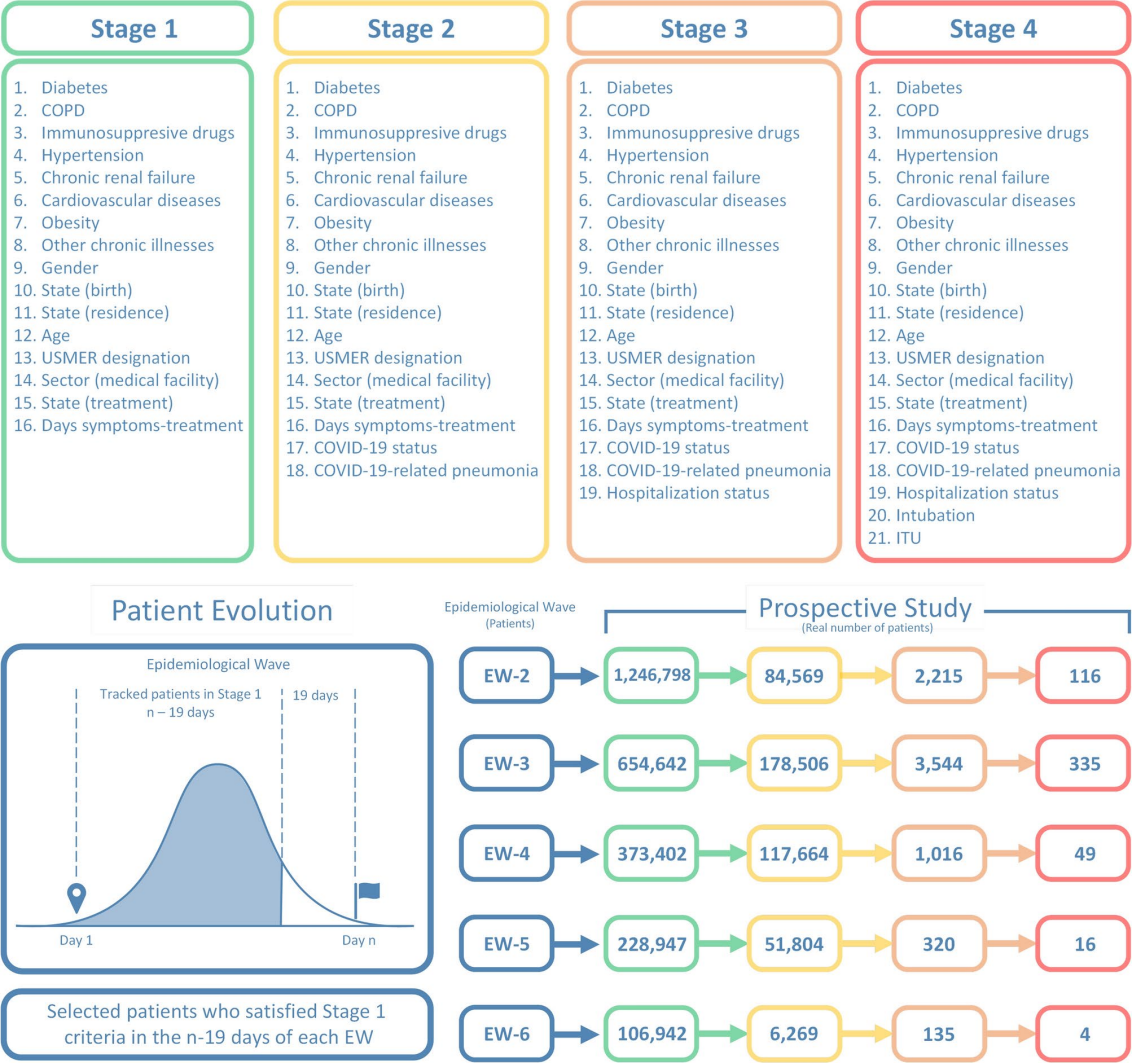
The top panels present the four clinical stages that were identified in Ref.^17^, for each of which we train a separate neural network designed to identify high-risk COVID patients. The flow diagram in the bottom-left panel illustrates the possible clinical history of a particular patient, for each of waves EW-2 to EW-6, ranging from Day 1 to the last day the wave in question. The bottom-right panel presents the number of identified patients obtained from our patient tracking protocol in each combination of clinical stage and epidemiological wave.

As described in Ref.^17^, each patient profile in the database comprises 28 characteristics. As outlined in Ref.^17^, each patient profile in the database includes 28 characteristics, encompassing medical history, demographic details, and recent COVID-19-related medical information. However, the authors excluded 7 of these characteristics due to their limited predictive power, inconsistent results stemming from small sample sizes, or weak correlations with outcomes. The excluded characteristics were pregnancy, asthma, smoking, indigenous status, indigenous language, migrant status, and foreign status^35^. Following the selection of the 21 statistically representative characteristics, the data underwent preprocessing. This involved numeric encoding, which converts categorical data into numeric values, followed by min-max normalization of the resulting numeric data. Consequently, in our analysis the resulting input vector for neural networks comprised 21 features, categorized into three groups: (1) medical history, (2) demographic data, and (3) recent medical information. Category 1 includes diabetes, chronic obstructive pulmonary disease (COPD), use of immunosuppressive drugs, hypertension, chronic renal failure, and cardiovascular diseases. Category 2 includes gender, age, state of birth, state of residence, and age. Category 3 comprises health monitoring units of viral respiratory disease (USMER) designation, type of healthcare facility where the patient is receiving treatment, state of treatment, number of days between the onset of symptoms and the beginning of treatment, COVID-19 status, COVID-19-related pneumonia, hospitalization status, intubation and admission into an intensive care unit (ICU). From these three categories, four clinical stages of the treatment process can be identified^17^, ranging from stage 1, at which the patient first seeks medical care, to stage 4, at which the patient is hospitalized and requires specialized medical care. Note that the outcome of each treatment stage, i.e. recovered patient or deceased patient, serves as the criterion for assessing the predictive accuracy of the neural networks.

In this prospective study, we conduct patient tracking through each epidemiological wave to assess the predictive capabilities of the models proposed by Ref.^17^. It is worth pointing out that those models were trained using data covering the period up to 31 January 2021. As of the present writing, it has the following attributes:

- **Recorded dates. ** The database covers the period from May 12 2020 to April 4 2023. In this period, it contains the historical record of 25,118,719 patients. Figure 1(a) is a summary of the COVID-19 outbreak in Mexico. Three statistical indicators are provided for each wave that delineate the progression of the pandemic in Mexico: the cumulative epidemic percentage (CEP), the epidemic wave percentage (EWP), and the cumulative percentage in the Mexican population (CPM). CEP denotes the proportion of confirmed COVID-19 cases at that specific time. EWP represents the ratio of confirmed cases from each epidemiological wave to the total confirmed cases in Mexico, whereas CPM indicates the progression of confirmed cases over time relative to the total population of Mexico.
- **Epidemiological waves.** During the pandemic Mexico officially experienced six epidemiological waves (EWs) with variable duration and four inter-pandemic periods (IPs). Figure 1(a) illustrates each EW in blue, while IPs are highlighted in gray. The corresponding periods and duration, expressed in weeks for both, EWs and IPs, are presented in Table 1.
- **Database sampling.** The database contains daily record updates up to the period IP-4. Unfortunately, in the beginning of this inter-pandemic period, the sampling frequency became irregular—attributed to the low prevalence of cases—until eventually updates to the database ceased. This implies that when carrying out a prospective study for patients in EW-5, there is uncertainty as to the specific day when a patient transitions from one stage to another.
- **Survivors and Death Tolls.** As of the date of the last update to the database, 456,252 deaths had been recorded among the 25,118,719 patients included in the historical database. Figure 1b shows a cumulative histogram depicting the total number of deaths experienced within each epidemiological wave during the entire COVID-19 outbreak.

It is important to comment on the COVID-19 vaccination campaign in Mexico, which formally started on 24 december 2020. The latest vaccination record is dated 7 October 2022, indicating that by that date 76.04% of the Mexican population had received at least one vaccination dose. Thus, in the period encompassed between midway through EW-2 (when the campaign started) and the end of EW-5, slightly more than three-quarters of the population received a vaccination^36^. By examining this information, one can identify specific dates corresponding to significant percentages within the vaccinated population. From the total vaccinated population up to the date of this study, 25%, 50%, and 75% received their vaccinations on June 7, 2021, August 4, 2021, and October 29, 2021, respectively. Figure  1a illustrates these dates with red rectangles, along with the cumulative percentage of the country’s population that has received at least one vaccine dose.

**Table 2:** Prospective overall accuracy of early-data-trained neural networks.

Wave	Stage			
	1	2	3	4
EW-2	80.19%	88.55%	70.61%	81.90%
EW-3	88.90%	94.36%	74.01%	84.79%
EW-4	80.21%	93.96%	71.75%	85.71%
EW-5	88.03%	93.71%	80.94%	62.50%
EW-6	87.41%	89.78%	68.15%	75.00%

The networks, described in Ref.^17^, are tested with data obtained from the patient tracking process for each of the four clinical stages within every epidemiological wave.

### Clinical stages

In our study, we have used the same four clinical stages of the treatment process that were identified in Ref.^17^. Each stage includes a specific number of characteristics, as illustrated in the top panels of Fig. 2. Stage 1 involves patients undergoing an initial medical evaluation and/or treatment. In Stage 2, patients have a confirmed COVID-19 status as part of the medical evaluation, and may already present COVID-19-related pneumonia. The patients in stage 3 have either already been admitted to a hospital, or have returned home after a hospital stay; patients in Stage 4 are those who have been intubated or admitted into an ICU unit. This categorization into clinical stages led to the training of four separate neural networks, one for each stage, which are discussed below.

### Healthcare system coverage limitations

In September 2023, the Government of Mexico, via the General Directorate of Epidemiology, released the document entitled “Standardized Guidelines for Epidemiological and Laboratory Surveillance of Viral Respiratory Disease.”^37^. The document outlines the essential protocols and standards for monitoring infectious diseases, including those used during the COVID-19 outbreak, influenza, and its variants. Based on its content, we have identified potential limitations and critical issues within epidemiological monitoring systems, which we recognize as constraints of our study. First, the surveillance strategy relies on 464 Viral Respiratory Disease Monitoring Health Units (USMER, by its Spanish initials). Although these units are strategically distributed, they may not ensure equitable coverage across all regions, particularly in rural or remote areas with limited access to healthcare services. Furthermore, the capacity of these units to handle a sudden surge in service demand is not addressed, which could impact both coverage and quality of care during high transmission periods. This limitation may result in an underestimation of cases in certain regions, thereby constraining the ability of models to generate generalizable predictions.

Second, the accuracy of case detection depends on symptom presentation and sample collection for laboratory testing (RT-PCR). In this regard, it does not acknowledge the potential for false negatives or false positives, which could compromise surveillance accuracy. False negatives may result from inadequate sample collection, low viral load during early or late stages of infection, or technical limitations of diagnostic tools. Notably, sampling is conducted on 10% of outpatient cases and 100% of inpatient cases, thereby excluding a significant proportion of mild or asymptomatic infections. Since these cases are critical for understanding community transmission and disease dynamics, this approach may lead to an underestimation of the virus’s true spread within the population. Another inherent limitation is the reliance on the timely reporting of suspected and confirmed cases. While the document emphasizes the importance of reporting within 24 hours, it does not address the handling of reporting delays, non-reporting, or potential underreporting, particularly in healthcare facilities with limited staff or resources. These delays or omissions could compromise the accuracy of epidemiological data.

Finally, although the document underscores the importance of training healthcare personnel in sample collection, case reporting, and management, it does not specify whether all levels of the healthcare system, particularly in resource-limited regions, have access to this training or if there are gaps in the implementation of protocols. Insufficient training or resource availability in certain areas may compromise data quality due to inadequate oversight and adherence to established protocols.

### Neural network

Supervised machine learning provides computer algorithms with the ability to *learn* from a known dataset to identify features and generate predictions about the outcome given a specific set of features, not included in the learning stage. In this context, using the publicly available database of Mexican COVID-19 patients, reference^17^ reported on an artificial neural network capable of classifying patients into two mortality-based classes: (a) recovered patient or (b) deceased patient. To assess the predictive accuracy of these neural networks over several COVID-19 waves, we conducted a prospective study organized into two distinct phases. In the first phase, we utilized the neural networks trained in Ref.^17^ for each of the four clinical stages. We then assessed their performance when applied to patients from epidemiological waves occurring after the dates of the data used for training. These patients were identified through a patient-tracking protocol within each of the epidemiological waves. In the second phase, we trained new neural networks using cumulative historical data up to the end of each wave and evaluated them with the same tracked patients from the previous phase. To ensure a fair comparison, the neural network architecture was identical to that reported in Ref.^17^ in all cases.

Importantly, all of our neural networks rely on a feed-forward architecture with two layers. The hidden and output layers comprise two sigmoid neurons and two softmax neurons, respectively. Importantly, the hyperparameters in the models were manually tuned using a trial-and-error approach, where the outcomes of each trial were analyzed and used to iteratively refine the hyperparameters in order to maximize model performance. While this method has proven effective in identifying optimal configurations, it can also be time-consuming^38–40^. Cross-entropy was selected as the training cost^41–43^, and the scaled conjugate gradient back-propagation method was used as optimizer^44,45^.

## Methodology

Similar techniques employing machine learning tools have been utilized to facilitate the monitoring of high-risk COVID-19 patients. Chenxi Sun^46^ offers a model employing a time-aware long short-term memory (T-LSTM) neural network, utilizing a database of patient blood samples gathered over a 5-week duration. The database comprises 80 features, of which 40 are biomarkers, including Lymph, LDH, hs-CRP, Indirect Bilirubin, and Creatinine. Mohammad M. Banoei^47^ employs a mortality prediction model for high-risk patients utilizing a statistically inspired modification of the partial least squares (SIMPLS) model, applied to a dataset of 250 parameters related to patients’ demographics, clinical factors, and comorbidities. Jun Wang^48^ utilizes a model based on a 3D convolutional neural network to forecast the recovery duration of COVID-19 patients. The model is supplied with a database including 2,530 patient records that include computed tomography scans, treatment plans, clinical factors, sociodemographic information, and patient symptoms. The referenced approaches are significantly reliant on the resources accessible for the specific study region and their inherent characteristics, complicating direct comparisons among them.

Our methodology employs a compact feed-forward neural network model to predict the mortality of high-risk COVID-19 patients, utilizing a database comprised solely of sociodemographic information and comorbidities of all patients in Mexico identified as infected with the SARS-CoV-2 virus, as delineated in^37^. Before initiating any phase of our prospective study, we have first conducted a patient-tracking protocol within each of the epidemiological waves, commencing with EW-2. The database analysis revealed that the mean incubation period was approximately 19 days. So, in order to consider as many patients as possible in the tracking protocol in each wave, we identify patients, who meet the criteria for stage 1, in any of the first $(n-19)$ days of each wave, i.e. from day 1 up to day $(n-19)$. Here, *n* corresponds to the duration in days of each wave. In stage 1, these criteria correspond to those who seek medical evaluation and/or treatment and are suspected of harboring a COVID-19 infection, as yet unconfirmed either through a test or the appearance of COVID-19-related pneumonia symptoms. Note that this patient tracking procedure excludes any patients who may fall ill later than day $(n-19)$ of each wave. Selected patients are then monitored day by day, until reaching the last day of the wave, registering any patients who may transition to stages 2, 3, and 4. It is important to note that we only monitor transitions that involve an upward stage change, i.e. stage 1 to stage 2, stage 2 to stage 3, and stage 3 to stage 4. Note that patients in Stage 2 already have a defined COVID-19 status and may present COVID-19-related pneumonia symptoms, so the list of features is expanded accordingly. Those patients who transition to Stage 3 have been hospitalized, activating the hospitalization status feature. Patients in Stage 4 are those who unfortunately reach a critical state and have undergone intubation or have been admitted to an ICU unit, resulting in the activation of the corresponding features in their profiles.

As part of the first phase of our prospective study, we utilized the neural network described in Ref.^17^ to assess its performance using patient data obtained during the tracking protocol. In the second phase, we trained new neural networks for each epidemiological wave and their respective clinical stages. These neural networks were trained with cumulative historical data up to the end of each wave, resulting in the training of twenty neural networks-five sets (EW-2 to EW-6) of four neural networks (one for each clinical stage). Each set of neural networks was evaluated using the same patient data obtained during the tracking protocol. This approach was designed to assess how responsive mortality prediction is to the number of epidemiological waves used in the training process and to forecast the likelihood of death for each clinical stage, both in the training epidemiological waves and in future waves.

**Table 3:** Overall accuracy of the five sets of neural networks trained with cumulative historical data from each epidemiological wave at different clinical stages.

Training Data	Stage			
	1	2	3	4
Ref.^17^ (January 31, 2021)	84.3%	90.5%	93.1%	93.5%
EW-2 (up to week 15 of 2021)	84.8%	91.0%	94.2%	94.2%
EW-3 (up to week 42 of 2021)	85.1%	91.6%	95.0%	95.1%
EW-4 (up to week 9 of 2022)	87.7%	91.6%	95.0%	95.1%
EW-5 (up to week 33 of 2022)	85.1%	91.6%	96.3%	96.4%
EW-6 (up to week 4 of 2023)	86.1%	91.5%	96.3%	95.5%

**Fig. 3:**
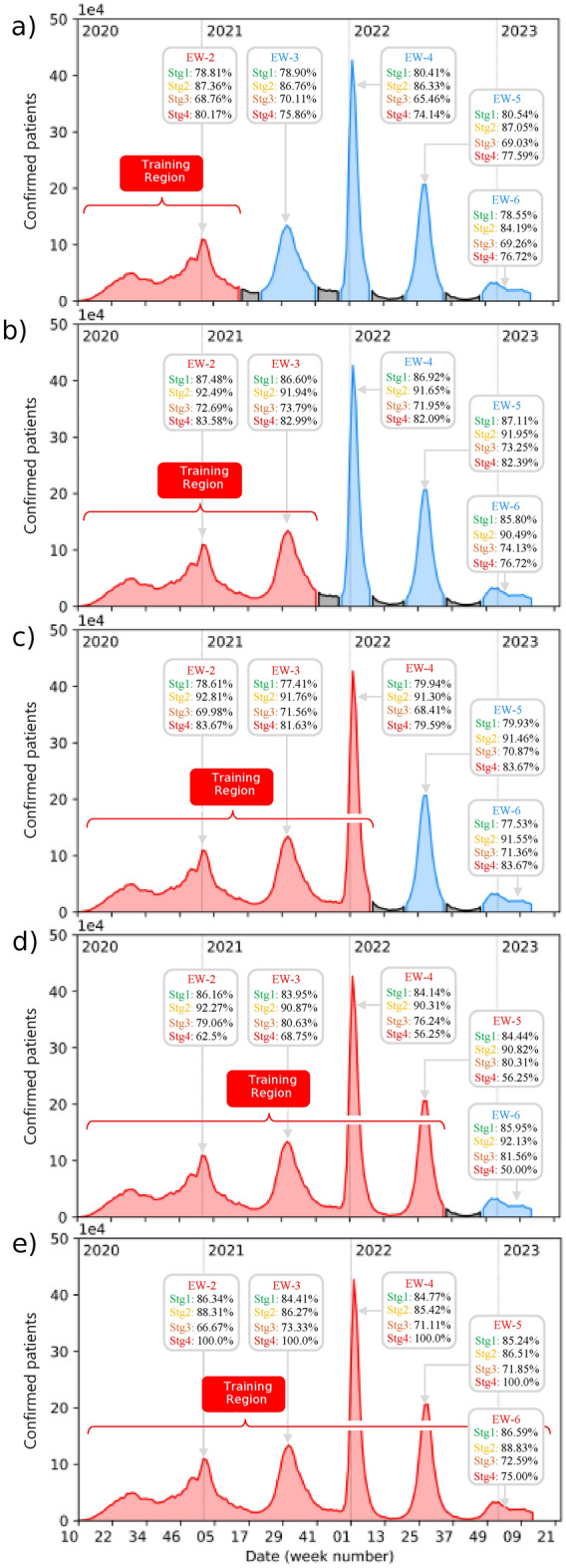
Prediction accuracy of our neural networks, calculated for patients in each of waves EW-2 through EW-6, for each of the four clinical stages. The length of the training period is progressively extended, adding one wave at a time, as displayed in the five subpanels so that in (e) data from all waves is used for training.

**Fig. 4:**
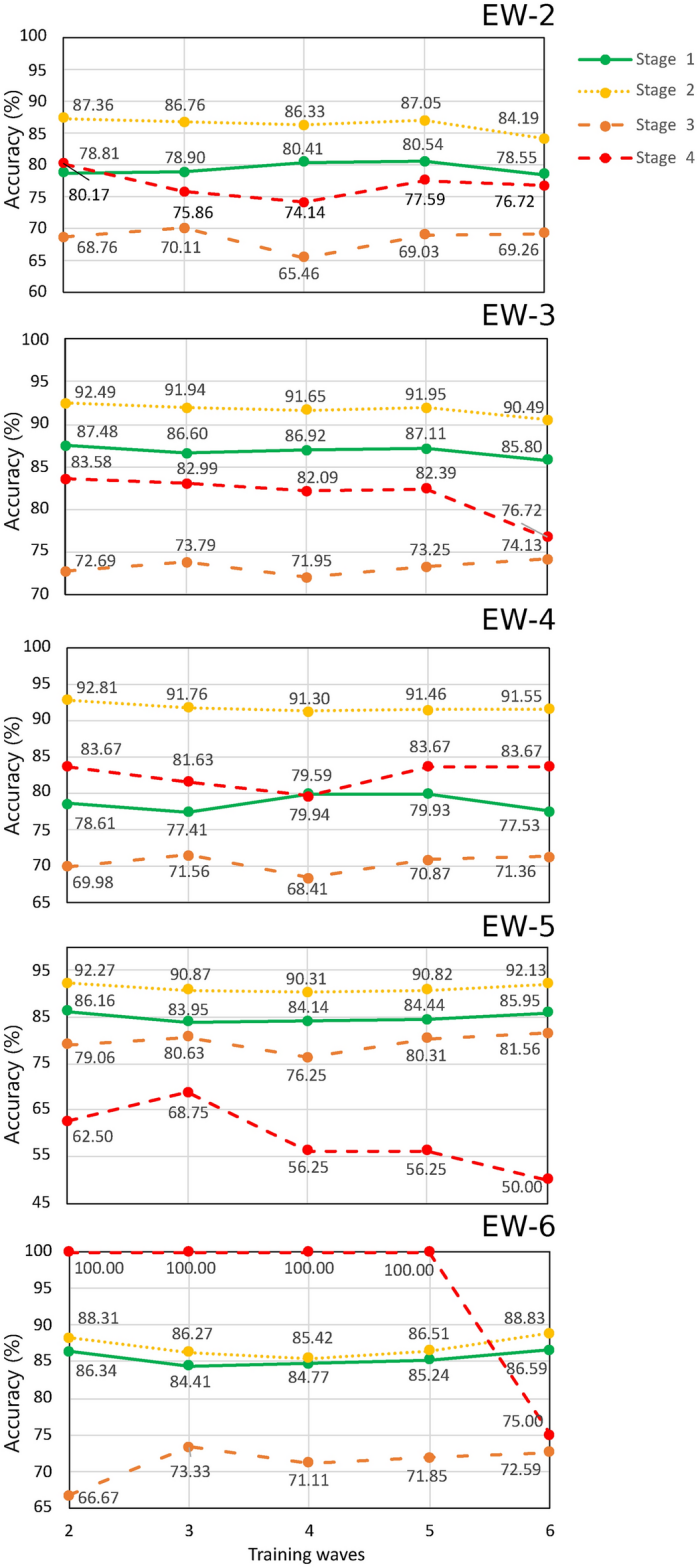
Prediction accuracy plotted, for each of stages 1–4 as labeled, vs the length of the training data date span, indicated in the horizontal axis as the most recent wave included. The accuracy values are obtained upon applying our neural networks to testing data resulting from our patient protocol tracking protocol; each subpanel indicates the use of data corresponding to each of waves EW-2 through EW-6.

These networks are tested during the second phase of the prospective study, see Figs. 3 and 4.

## Results

In this section, we present the results of our patient-tracking protocol as well as the two stages of our prospective study. As part of our tracking protocol, we show in the bottom panel of Fig. 2, the number of patients who start off at stage 1 in each wave (EW-2 through EW-5), as well as the number transitioning to each of stages 2, 3, and 4. Despite the severity of the pandemic, the number of patients transitioning to higher stages is relatively low. Fortunately, most patients do not suffer complications and remain at stage 1 until discharge. Since our patient-tracking protocol begins by identifying patients at stage 1 within the first $n-19$ days (excluding those who started at higher stages) and the transition probability to higher stages is low, the number of patients reaching stage 4 is quite small.

Note that our patient tracking protocol described above reflects the sequence of events that could be followed by a particular patient. Upon arrival of the patient at a clinic or hospital they are inspected at the triage, and from there transitions through some or all of the four clinical stages, with either recovery or death as the final outcome. It is at the triage stage that a predictive tool such as our neural networks becomes relevant: it can aid healthcare professionals to identify patients who are at higher risk, thus helping to more efficiently manage the hospital resources and capacity, and to provide timely treatment. In this regard, we utilized the neural networks trained in Ref.^17^ to assess their predictive power using the patient data obtained during the tracking process. The resulting accuracy of these neural networks at each clinical stage and each epidemiological wave is recorded in Table 2. The accuracy is calculated as the sum of true negatives and true positives divided by the total number of records.

Because the networks from Ref.^17^ were trained using data covering the period up to 31 January 2021, we have tested them on patients ranging from waves EW-2 to EW-6. The accuracies shown in Table 2, which correspond to Stages 1 and 2, exhibit high efficiencies due to the large number of records in these stages. Stage 3 does not contain a sufficient number of records for the neural networks to detect patterns correctly, in particular for waves EW-5 and EW-6, which leads to a markedly lower accuracy. This difficulty is compounded for stage 4, which exhibits a very small number of cases leading to poor statistics and the inability to correctly estimate the algorithm efficiency.

**Table 4:** Overall accuracy results for different models trained across the four stages in EW-2.

Train	Test	RF	SVM	LR	NN
Stg1	W2_Stg1	60.99	99.42	64.81	78.81
	W3_Stg1	71.92	99.73	78.3	87.48
	W4_Stg1	62.57	99.72	68.78	78.61
	W5_Stg1	59.82	99.74	73.47	86.16
	W6_Stg1	65.70	99.72	73.56	83.34
Stg2	W2_Stg2	84.0	98.32	89.02	87.36
	W3_Stg2	90.48	99.14	93.53	92.49
	W4_Stg2	89.96	98.97	93.78	92.81
	W5_Stg2	86.68	99.42	92.98	92.27
	W6_Stg2	82.35	98.93	88.79	88.31
Stg3	W2_Stg3	51.64	80.63	52.23	68.63
	W3_Stg3	61.17	72.91	63.35	72.69
	W4_Stg3	60.64	78.66	60.67	69.98
	W5_Stg3	74.01	79.3	77.71	79.06
	W6_Stg3	71.46	73.08	70.77	66.67
Stg4	W2_Stg4	46.12	56.03	43.97	80.17
	W3_Stg4	53.71	53.69	57.82	83.58
	W4_Stg4	41.43	57.14	42.86	83.67
	W5_Stg4	45.0	50.0	56.25	62.5
	W6_Stg4	32.5	75.0	50.0	100.0

The models were tested on identified patients from our tracking protocol across their corresponding waves and stages.

**Table 5:** Overall accuracy results for different models trained across the four stages in EW-3.

Train	Test	RF	SVM	LR	NN
Stg1	W2_Stg1	74.38	99.45	64.05	78.98
	W3_Stg1	79.51	99.69	77.92	86.6
	W4_Stg1	71.30	91.06	68.19	77.41
	W5_Stg1	65.35	99.25	73.62	83.95
	W6_Stg1	79.45	99.47	73.56	84.41
Stg2	W2_Stg2	80.03	99.37	87.66	86.76
	W3_Stg2	88.86	99.81	92.61	91.94
	W4_Stg2	84.69	99.86	93.02	91.76
	W5_Stg2	84.05	99.93	92.26	90.87
	W6_Stg2	85.69	99.78	87.75	86.27
Stg3	W2_Stg3	51.60	80.95	51.69	70.11
	W3_Stg3	64.36	73.35	64.1	73.79
	W4_Stg3	62.36	79.15	61.26	71.56
	W5_Stg3	76.75	79.94	78.03	80.63
	W6_Stg3	73.54	72.31	70.77	73.33
Stg4	W2_Stg4	44.48	57.76	43.97	75.86
	W3_Stg4	54.63	39.53	59.88	82.99
	W4_Stg4	41.83	63.27	42.86	81.63
	W5_Stg4	50.0	50.0	56.25	68.75
	W6_Stg4	40.0	50.0	50.0	100.0

The models were tested on identified patients from our tracking protocol across their corresponding waves and stages.

In the second phase of our prospective study, we train five sets of neural networks using cumulative historical data covering the period up to the end of each wave (EW-2 through EW-5). We then evaluate their prediction effectiveness using data obtained from the patient tracking process. Through this study, we aim to ascertain the optimal number of waves to include in the training of neural networks for predicting high-risk COVID-19 patients with the highest accuracy possible.

We first describe how the second-phase neural networks are prepared. As was the case in the first phase of our study, we trained a separate network for each of the four stages. The first set of four networks is trained with data covering the period up until the end of EW-2, i.e. week 15 of 2021. The second set of four networks is trained with data covering the period up to the end of EW-3, i.e. week 42 of 2021. This process has been repeated for data covering the periods up until the end of EW-4, EW-5, and EW-6, corresponding to week 9 of 2022, week 33 of 2022, and week 13 of 2023, respectively. Following previous work^17^, we allocate 70% of the data for training, 15% for validation, and 15% for testing. The accuracy of each set of neural networks is shown in Table 3.

To assess the predictive capabilities of our trained network set, we have tested it on the dataset obtained through the patient tracking protocol, for each wave, used in the first phase of our study. In Fig. 3 we plot the number of new confirmed patients plotted vs date throughout the date span covered by the database, as was also done in Fig, 1(a). For each wave EW-2 through EW-5, and each of the four stages, we display the resulting accuracies of our networks in the gray boxes. In the five subpanels, (a) through (e), we employ for neural network training a progressively larger fraction of the total date span, colored in red (note that we have colored in blue the excluded data). Note that despite the enlargement of the training region, the accuracies remain remarkably consistent in all cases. More importantly, these accuracies are comparable to those achieved with the neural network proposed in Ref.^17^. These findings suggest that neural networks trained with historical data during the early stages of the pandemic exhibit strong predictive capabilities, which permits precise identification of high-risk patients in subsequent epidemiological waves.

**Table 6:** Overall accuracy results for different models trained across the four stages in EW-4.

Train	Test	RF	SVM	LR	NN
Stg1	W2_Stg1	77.82	90.76	63.93	80.41
	W3_Stg1	79.44	94.78	77.2	86.92
	W4_Stg1	75.85	93.32	67.07	79.59
	W5_Stg1	76.91	92.76	70.75	84.14
	W6_Stg1	72.64	89.28	70.68	84.77
Stg2	W2_Stg2	80.48	99.53	86.9	86.33
	W3_Stg2	88.19	99.89	92.03	91.65
	W4_Stg2	87.54	99.96	92.66	91.3
	W5_Stg2	84.50	99.98	92.12	90.3
	W6_Stg2	82.68	99.98	87.75	85.42
Stg3	W2_Stg3	50.44	80.99	50.74	65.46
	W3_Stg3	62.60	73.3	63.3	71.95
	W4_Stg3	57.45	79.06	61.26	68.41
	W5_Stg3	76.59	79.62	77.71	76.25
	W6_Stg3	72.77	72.31	71.54	71.11
Stg4	W2_Stg4	43.01	53.45	43.1	74.14
	W3_Stg4	58.23	55.16	60.18	82.09
	W4_Stg4	42.86	57.14	44.9	79.94
	W5_Stg4	43.12	50.0	56.25	56.25
	W6_Stg4	40.0	75.0	50.0	100.0

The models were tested on identified patients from our tracking protocol across their corresponding waves and stages.

**Table 7:** Overall accuracy results for different models trained across the four stages in EW-5.

Train	Test	RF	SVM	LR	NN
Stg1	W2_Stg1	75.40	99.09	64.73	80.54
	W3_Stg1	77.54	99.56	77.91	87.11
	W4_Stg1	79.68	99.55	68.0	79.93
	W5_Stg1	76.26	99.55	71.8	84.44
	W6_Stg1	75.63	99.62	71.76	85.24
Stg2	W2_Stg2	84.53	99.33	86.85	87.05
	W3_Stg2	87.31	99.76	92.02	91.95
	W4_Stg2	87.93	99.87	92.88	91.46
	W5_Stg2	91.17	99.92	92.89	90.82
	W6_Stg2	88.46	99.84	89.55	86.51
Stg3	W2_Stg3	48.57	58.01	49.93	69.03
	W3_Stg3	62.14	63.19	62.6	73.25
	W4_Stg3	64.60	55.56	61.06	70.87
	W5_Stg3	77.70	70.38	77.71	80.31
	W6_Stg3	71.15	66.92	71.54	71.85
Stg4	W2_Stg4	43.70	50.86	43.97	77.59
	W3_Stg4	60.23	57.82	60.18	82.39
	W4_Stg4	44.08	53.06	44.9	83.67
	W5_Stg4	49.37	56.25	56.25	56.25
	W6_Stg4	35.0	75.0	50.0	100.0

The models were tested on identified patients from our tracking protocol across their corresponding waves and stages.

For the sake of completeness, and to explicitly show the robustness of early-data-trained neural networks, Fig. 4 shows the accuracy of neural networks trained with increasingly larger datasets (including an increasing number of waves) when predicting patient clinical outcomes for patients in each of the stages, and in each of waves EW-2 through EW-6. Each subpanel, 4a–e, shows the prediction accuracies of the neural network, with a structure identical to that used in Ref.^17^, trained with data up to the epidemiological wave number indicated in the horizontal axis. The subpanel labels shown in the upper right corner denote the epidemiological waves to which the patient tracking protocol is applied to obtain the testing data. Note that Stage 4 (red line) of all waves presents slight fluctuations, which follow from the limited size of the respective dataset.

Finally, we remark that Fig. 4 allows for concluding that early-data-trained neural networks, such as the one used in Ref.^17^, can predict with a precision greater than 85.05% the mortality outcome for patients who are tracked since they enter in Stage 2, i.e., patients with a confirmed diagnosis of COVID-19 who do not yet present respiratory complications. The aforementioned results were obtained by training the neural network using data up until week 13 of 2021, corresponding to EW-2. This corresponds to 6.55% of the individuals who have been vaccinated, which is equivalent to 9.17% of the total Mexican population infected as of the date of the study. This may certainly allow for a more efficient allocation of resources for such patients.

Furthermore, we have trained Random Forest (RF), Support Vector Machine (SVM), and Logistic Regression (LR) models to compare the performance of our neural networks against other machine learning algorithms. We conducted ten independent realizations of each model to ensure statistical reliability. The overall accuracies reported represent the mean of these ten realizations. Tables 4, 5, 6, 7 and  8 show the results obtained from each model, including also the results from our Neural Network (NN).

**Table 8:** Overall accuracy results for different models trained across the four stages in EW-6.

Train	Test	RF	SVM	LR	NN
Stg1	W2_Stg1	77.68	99.8	69.0	78.55
	W3_Stg1	81.41	99.98	81.3	85.8
	W4_Stg1	76.52	99.79	73.05	77.53
	W5_Stg1	78.59	99.98	78.69	85.95
	W6_Stg1	78.11	99.97	78.53	86.59
Stg2	W2_Stg2	83.02	98.87	86.05	84.19
	W3_Stg2	88.62	99.47	91.53	90.49
	W4_Stg2	88.20	98.92	92.59	91.55
	W5_Stg2	89.27	99.67	92.84	92.13
	W6_Stg2	87.85	99.12	89.79	88.83
Stg3	W2_Stg3	47.47	28.17	48.58	69.26
	W3_Stg3	63.32	40.47	61.63	74.13
	W4_Stg3	60.68	34.81	59.59	71.36
	W5_Stg3	74.29	47.77	77.71	81.56
	W6_Stg3	73.23	51.54	71.54	72.59
Stg4	W2_Stg4	45.94	57.76	43.1	76.72
	W3_Stg4	56.07	37.46	59.0	76.72
	W4_Stg4	39.3	63.27	44.9	83.67
	W5_Stg4	49.37	43.75	56.25	50.0
	W6_Stg4	35.0	75.0	50.0	75.0

The models were tested on identified patients from our tracking protocol across their corresponding waves and stages.

To compare the overall performance of the trained models, we calculated the average accuracy across all waves and clinical stages. This general score allows for a comparison of each evaluated model. The resulting average accuracies were 67.53% for RF, 80.18% for SVM, 69.54% for LR, and 81.01% for NN. Notably, the NN and SVM exhibit similar performance, both surpassing that of the RF and LR models. RF achieves an accuracy of 80% for stage 2 patients across different training waves (see Tables 4, 5, 6, 7 and 8); however, its performance declines significantly in other stages. SVM demonstrates high predictive capability for mortality in stages 1 and 2 across training waves but exhibits a notable drop in accuracy for stage 4 patients and demands the highest computational resources among the four models. The performance of LR closely resembles that of RF, achieving over 86% accuracy for stage 2 patients. The proposed neural network model attains an accuracy exceeding 78% for stage 1, 85% for stage 2, 65% for stage 3, and 50% for stage 4. We believe that the results of this prospective study provide the building blocks for novel strategies to predict outcomes in clinical settings. Moreover, they provide new tools for clinical decision-making in the context of future large-scale epidemics or pandemic events. To ensure the reproducibility of this study, the codes for the patient-tracking protocol, as well as the preprocessing and training/testing models, are available in the GitHub repository at https://github.com/CFATA-AI/COVID-19.

### Adaptability of ML-based risk prediction across healthcare systems

While our study proposes a flexible AI-based methodology to identify high-risk COVID-19 patients for prioritized care, thereby optimizing resource allocation, several challenges must be addressed to ensure its effective application across diverse countries and health systems. Variations in healthcare infrastructure, such as the availability of centralized electronic health records, can significantly impact data collection and model implementation. In regions with limited electronic health records systems, the input feature vector must be adjusted to align with available data sources. Decentralized data collection methods, such as mobile health applications or community-based reporting systems, could serve as viable alternatives.

Data availability and quality also vary significantly across regions. In some areas, data can be fragmented, incomplete, or inconsistently recorded. To address these challenges, models can be trained with smaller and localized datasets and enhanced using techniques such as data augmentation or transfer learning. Additionally, sociodemographic characteristics-including age distribution, healthcare-seeking behavior, and cultural practices-can influence health outcomes and data collection. Integrating demographic stratification and behavioral data into the models can help identify the effect of these variations, improving predictive accuracy.

Another critical consideration is the variation in disease prevalence and associated risk factors across populations. Comorbidities or demographic variables that are significant predictors in one region may be less relevant in another. To ensure relevance and accuracy, models must be retrained with localized data and incorporate region-specific risk variables. Engaging local communities and stakeholders can further refine model performance by providing insights into culturally specific factors that impact healthcare access and outcomes. This approach is essential for tailoring the methodology to the unique epidemiological and sociodemographic profiles of different populations.

## Conclusions

Although the COVID-19 crisis has by now taken a backseat in current world affairs, it is of utmost importance to provide health-sector professionals with technological tools that allow them to effectively manage available clinical resources during possible future pandemics or large-scale epidemic events. In Ref.^17^ we proposed state-of-the-art machine-learning models that can, with high efficiency, identify high-risk COVID-19 patients across four clearly-identified clinical stages. In this work, we have presented a prospective validation study, based on tracking individual patients day-to-day in each of the latter five COVID-19 epidemiological waves, among the six officially recognized waves in Mexico. We apply our machine-learning models from Ref.^17^ to the patients in datasets obtained from our patient tracking protocol. On the one hand, we employ the same networks reported in^17^ (without any retraining), and apply them prospectively to patients in each of waves EW-2 through EW-6. On the other hand, we retrained the networks with data covering an increasing date span to include a successively larger number of epidemiological waves. We then applied prospectively these networks to patients in each of waves EW-2 through EW-6. Our results show that models trained with early historical data exhibit significant predictive capabilities permitting precise identification of high-risk patients in subsequent epidemiological waves, with efficiencies in line with those obtained with networks trained with data from an extended date span. We believe these results establish the grounds for innovative strategies in predicting individual clinical outcomes in the context of epidemiology, providing valuable insights for possible future health crises.

## Data Availability

The data that support the findings of this study are openly available in the Statistical Morbidity Yearbooks published by the General Council of Epidemiology, part of the Health Ministry, Mexican Federal Government. Data are available in https://www.gob.mx/salud/documentos/datos-abiertos-bases-historicasdireccion-general-de-epidemiologia.

## References

[1] Zhou, P. et al. A pneumonia outbreak associated with a new coronavirus of probable bat origin. *Nature* **579**, 270. 10.1038/S41586-020-2012-7 (2020). 32015507 10.1038/s41586-020-2012-7 PMC7095418

[2] Chavda, V. P., Patel, A. B. & Vaghasiya, D. D. SARS-CoV-2 variants and vulnerability at the global level. *J. Med. Virol.* **94**, 2986 (2022). 35277864 10.1002/jmv.27717 PMC9088647

[3] McKibbin, W. J. & Fernando, R. Macroeconomic policy adjustments due to COVID-19: scenarios to 2025 with a focus on Asia. *Soc. Sci. Res. Netw.* **2020**, 45. 10.2139/SSRN.3547729 (2020).

[4] Maital, S. & Barzani, E. The global economic impact of COVID-19: a summary of research. *Samuel Neaman Inst. National Policy Res.* **2020**, 1 (2020).

[5] Sharma, A., Borah, S. B. & Moses, A. Responses to COVID-19: the role of governance, healthcare infrastructure, and learning from past pandemics. *J. Bus. Res.* **122**, 597. 10.1016/J.JBUSRES.2020.09.011 (2020). 33518844 10.1016/j.jbusres.2020.09.011 PMC7834581

[6] Kaye, A. D. et al. Economic impact of COVID-19 pandemic on healthcare facilities and systems: international perspectives. *Best Pract. Res. Clin. Anaesthesiol.* **35**, 293 (2021). 34511220 10.1016/j.bpa.2020.11.009 PMC7670225

[7] Gray, D. M., Anyane-Yeboa, A., Balzora, S., Issaka, R. B. & May, F. P. COVID-19 and the other pandemic: populations made vulnerable by systemic inequity. *Nat. Rev. Gastroenterol. Hepatol.* **17**, 520 (2020). 32541960 10.1038/s41575-020-0330-8 PMC7294516

[8] Acuña-Zegarra, M. A., Santana-Cibrian, M. & Velasco-Hernandez, J. X. Modelo con estructura social para el estudio de medidas de control de la pandemia de COVID-19. *Math. Biosci.* **2020**, 108370 (2020). 10.1016/j.mbs.2020.108370 PMC7202859 32387384

[9] Kurkina, E. S. & Koltsova, E. M. Mathematical modeling of the propagation of Covid-19 pandemic waves in the. *Comput. Math. Model.* **32**, 147. 10.1007/S10598-021-09523-0 (2021).

[10] Jin, C. et al. Development and evaluation of an artificial intelligence system for COVID-19 diagnosis. *Nat. Commun.* **11**, 1 (2020). 33037212 10.1038/s41467-020-18685-1 PMC7547659

[11] Mei, X. et al. Artificial intelligence–enabled rapid diagnosis of patients with COVID-19. *Nat. Med.* **26**, 1224 (2020). 32427924 10.1038/s41591-020-0931-3 PMC7446729

[12] Harmon, S. A. et al. Artificial intelligence for the detection of COVID-19 pneumonia on chest CT using multinational datasets. *Nat. Commun.* **11**, 1 (2020). 32796848 10.1038/s41467-020-17971-2 PMC7429815

[13] Shi, F. et al. Review of artificial intelligence techniques in imaging data acquisition, segmentation, and diagnosis for COVID-19. *IEEE Rev. Biomed. Eng.* **14**, 4 (2020). 10.1109/RBME.2020.2987975 32305937

[14] Keshavarzi, A. et al. Artificial intelligence for COVID-19 drug discovery and vaccine development. *Front. Artif. Intell.* **3**, 65 (2020). 33733182 10.3389/frai.2020.00065 PMC7861281

[15] Vaishya, R., Javaid, M., Khan, I. H. & Haleem, A. Artificial Intelligence (AI) applications for COVID-19 pandemic. *Diabetes Metabol. Syndrome Clin. Res. Rev.* **14**, 337 (2020). 10.1016/j.dsx.2020.04.012 PMC7195043 32305024

[16] Rao, A. S. S. et al. Identification of COVID-19 can be quicker through artificial intelligence framework using a mobile phone–based survey when cities and towns are under quarantine. *Infect. Control Hospital Epidemiol.* **41**, 826 (2020). 10.1017/ice.2020.61 PMC7200852 32122430

[17] Quiroz-Juárez, M. A. et al. Identification of high-risk COVID-19 patients using machine learning. *PLoS ONE* **16**, e0257234 (2021). 34543294 10.1371/journal.pone.0257234 PMC8452016

[18] Muhammad, L. et al. Supervised machine learning models for prediction of COVID-19 infection using epidemiology dataset. *SN Comput. Sci.* **2**, 1 (2021). 10.1007/s42979-020-00394-7 PMC7694891 33263111

[19] Prieto, K. Current forecast of COVID-19 in Mexico: a Bayesian and machine learning approaches. *PLoS ONE* **17**, e0259958 (2022). 35061688 10.1371/journal.pone.0259958 PMC8782335

[20] Iwendi, C., Huescas, C., Chakraborty, C. & Mohan, S. COVID-19 health analysis and prediction using machine learning algorithms for Mexico and Brazil patients. *J. Exp. Theor. Artif. Intell.* **1**, 35 (2022).

[21] Martinez-Velazquez, R. et al. A machine learning approach as an aid for early COVID-19 detection. *Sensors* **21**, 4202 (2021). 34207437 10.3390/s21124202 PMC8235359

[22] Pradhan, A., Prabhu, S., Chadaga, K., Sengupta, S. & Nath, G. Supervised learning models for the preliminary detection of COVID-19 in patients using demographic and epidemiological parameters. *Information* **13**, 330 (2022).

[23] Maouche, I. et al. In * The Proceedings of the International Conference on Smart City Applications* 507–517 (Springer, 2021).

[24] De Souza, F. S. H., Hojo-Souza, N. S., Dos Santos, E. B., Da Silva, C. M. & Guidoni, D. L. Predicting the disease outcome in COVID-19 positive patients through Machine Learning: a retrospective cohort study with Brazilian data. *Front. Artif. Intell.* **4**, 579931 (2021). 34514377 10.3389/frai.2021.579931 PMC8427867

[25] Assaf, D. et al. Utilization of machine-learning models to accurately predict the risk for critical COVID-19. *Intern. Emerg. Med.* **15**, 1435 (2020). 32812204 10.1007/s11739-020-02475-0 PMC7433773

[26] Ahamad, M. M. et al. machine learning model to identify early stage symptoms of SARS-Cov-2 infected patients. *Expert Syst. Appl.* **160**, 113661 (2020). 32834556 10.1016/j.eswa.2020.113661 PMC7305929

[27] Nemati, M., Ansary, J. & Nemati, N. Machine-learning approaches in COVID-19 survival analysis and discharge-time likelihood prediction using clinical data. *Patterns* **1**, 100074 (2020). 32835314 10.1016/j.patter.2020.100074 PMC7334917

[28] Bezzan, V. P. & Rocco, C. D. Predicting special care during the COVID-19 pandemic: a machine learning approach. *Health Inf. Sci. Syst.* **9**, 34 (2021). 34413974 10.1007/s13755-021-00164-6 PMC8363860

[29] Subudhi, S. et al. Comparing machine learning algorithms for predicting ICU admission and mortality in COVID-19. *NPJ Digital Med.* **4**, 87 (2021). 10.1038/s41746-021-00456-x PMC8140139 34021235

[30] Pourhomayoun, M. & Shakibi, M. Predicting mortality risk in patients with COVID-19 using machine learning to help medical decision-making. *Smart Health* **20**, 100178 (2021). 33521226 10.1016/j.smhl.2020.100178 PMC7832156

[31] Epstein, J. M., Parker, J., Cummings, D. & Hammond, R. A. Coupled contagion dynamics of fear and disease: mathematical and computational explorations. *PLoS ONE* **3**, e3955 (2008). 19079607 10.1371/journal.pone.0003955 PMC2596968

[32] Jain, K., Bhatnagar, V., Prasad, S. & Kaur, S. Coupling fear and contagion for modeling epidemic dynamics. *IEEE Trans. Netw. Sci. Eng.* **10**, 20 (2022).

[33] Yu, J. & Zhao, J. Prediction of systemic risk contagion based on a dynamic complex network model using machine learning algorithm. *Complexity* **2020**, 1 (2020).

[34] de Epidemiología, D. G. Anuario estadísticos de morbilidad. https://www.gob.mx/salud/documentos/datos-abiertos-152127 (2023).

[35] Wetschoreck, F. *Towards Data Science* (Springer, 2020).

[36] Mathieu, E. et al. A global database of COVID-19 vaccinations. *Nat. Hum. Behav.* **5**, 947 (2021). 33972767 10.1038/s41562-021-01122-8

[37] de Epidemiología, D. G. Lineamiento estandarizado para la vigilancia epidemiológica y por laboratorio de la enfermedad respiratoria viral (2023, accessed 30 Jan 2025). https://www.gob.mx/salud/documentos/lineamiento-estandarizado-para-la-vigilancia-epidemiologica-y-por-laboratorio-de-la-enfermedad-respiratoria-viral.

[38] Hutter, F., Kotthoff, L. & Vanschoren, J. *Automated Machine Learning: Methods, Systems, Challenges* (Springer, 2019). 10.1007/978-3-030-05318-5.

[39] Liao, L. et al. An empirical study of the impact of hyperparameter tuning and model optimization on the performance properties of deep neural networks. *ACM Trans. Softw. Eng. Methodol.* **31**, 1 (2022).

[40] Ilemobayo, J. A. et al. Hyperparameter tuning in machine learning: a comprehensive review. *J. Eng. Res. Rep.* **26**, 388 (2024).

[41] De Boer, P.-T., Kroese, D. P., Mannor, S. & Rubinstein, R. Y. A tutorial on the cross-entropy method. *Ann. Oper. Res.* **134**, 19 (2005).

[42] Mannor, S., Peleg, D. & Rubinstein, R. In *Proceedings of the 22nd international conference on Machine learning* 561–568 (2005).

[43] Shore, J. & Johnson, R. Properties of cross-entropy minimization. *IEEE Trans. Inf. Theory* **27**, 472 (1981).

[44] Møller, M. F. A scaled conjugate gradient algorithm for fast supervised learning. *Neural Netw.* **6**, 525 (1993).

[45] Johansson, E. M., Dowla, F. U. & Goodman, D. M. Backpropagation learning for multilayer feed-forward neural networks using the conjugate gradient method. *Int. J. Neural Syst.* **2**, 291 (1991).

[46] Sun, C., Hong, S., Song, M., Li, H. & Wang, Z. Predicting COVID-19 disease progression and patient outcomes based on temporal deep learning. *BMC Med. Inf. Decis. Mak.* **21**, 1 (2021). 10.1186/s12911-020-01359-9 PMC7869774 33557818

[47] Banoei, M. M., Dinparastisaleh, R., Zadeh, A. V. & Mirsaeidi, M. Machine-learning-based COVID-19 mortality prediction model and identification of patients at low and high risk of dying. *Crit. Care* **25**, 1 (2021). 34496940 10.1186/s13054-021-03749-5 PMC8424411

[48] Wang, J. et al. iCOVID: interpretable deep learning framework for early recovery-time prediction of COVID-19 patients. *NPJ Digit. Med.* **4**, 124 (2021). 34400751 10.1038/s41746-021-00496-3 PMC8367981

